# Reliability and Validity Evidence of Scores on the French Version of the Questionnaire about Interpersonal Difficulties for Adolescents

**DOI:** 10.5334/pb.bl

**Published:** 2015-10-14

**Authors:** Mandarine Hugon, Beatriz Delgado, Candido J. Ingles, Maria D. Hidalgo, Jose M. Garcia-Fernandez, Maria C. Martinez-Monteagudo

**Affiliations:** 1 Laboratory of Developmental Psychology and Socialization Processes, University of Toulouse, France; 2 Department of Developmental Psychology and Didactics, University of Alicante. PO Box 99, 03080 Alicante, Spain; 3 Department of Health Psychology, Miguel Hernandez University of Elche, Spain; 4 Department of Basic Psychology and Methodology, University of Murcia, Spain

**Keywords:** Adolescence, Interpersonal anxiety, Social fears, Self-report, French validation

## Abstract

This study examined the reliability and validity evidence drawn from the scores of the French version of the Questionnaire about Interpersonal Difficulties for Adolescents (QIDA) in a sample of 957 adolescents (48.5% boys) ranging in age from 11 to 18 years (*M* = 14.48, *SD* = 1.85). A principal axis factoring (PAF) and confirmatory factor analyses (CFA) were performed to determine the fit of the factor structure of scores on the QIDA. PAF and CFA replicated the previously identified correlated five-factor structure of the QIDA: Assertiveness, Heterosexual Relationships, Public Speaking, Family Relationships, and Close Friendships. The QIDA yielded acceptable reliability scores for French adolescents. Validity evidence of QIDA was also established through correlations with scores on the School Anxiety Inventory and the Social Anxiety Scale for Adolescents. Most of the correlations were positive and exceeded the established criteria of statistical significance, but the magnitude of these varied according to the scales of the QIDA. Results supported the reliability and validity evidence drawn from the scores of the French version of the QIDA.

Adolescence is a developmental stage characterised by important changes in social relationships. New social demands (e.g., asking for a date, presenting oral communications in class, attending parties and dances) require adolescents to show greater independence in their social interactions, and this favors the onset of interpersonal anxiety and social fears in those young people who find social interactions more difficult. Impairments and anxiety in social relationships are a common problem during adolescence (Detweiler, Comer, & Albano, 2010; Ollendick, Benoit, & Grills-Taquechel, 2014; Wittchen, Stein, & Kessler, 1999), and as such, they are related to many problems including drug consumption (Wittchen et al., 1999), social rejection and isolation (Inglés, Delgado, García-Fernández, Ruíz-Esteban & Díaz-Herrero, 2010), negative self-concept and low self-esteem (Delgado, Inglés & García-Fernández, 2013; Rivers et al., 2012), poor academic achievement and early dropout (Bernstein, Bernat, Davis & Layne 2008; Delgado, Inglés & García-Fernández, 2014) as well as several emotional and anxiety disorders (Chartrand, Cox, El-Gabalawy & Clara, 2011; Bernstein et al., 2008; Wittchen et al., 1999).

According to the interpersonal model of social anxiety, interpersonal difficulties of socially anxious individuals are the primary maintainer of the problem, because avoidance and safety behaviours slow down the formation of social relationships and the ability to connect significantly with others (Alden, 2001; Wong, Gordon, & Heimberg, 2014). In addition, interpersonal anxiety shows a chronic course and is relatively stable when it is not treated (e.g., Carballo et al., 2010) and significantly interferes with the personal and social functioning of adolescents due to the deep distress and unease that it generates (Beidel et al., 2007). For these reasons, the assessment of anxiety in social situations during adolescence has garnered considerable attention (see Tulbure, Szentagotai, Dobrean, & David, 2012, for a review).

Despite this fact, there is a lack of psychometrically sound self-report measures to assess interpersonal anxiety identifying different contexts and social behaviors in French adolescents. Currently, there are some measures of social anxiety in French language such as the Liebowitz Social Anxiety Scale (Schmits, Heeren, & Quertemont, 2014), the Fear of Negative Evaluation Scale (Monfette, Grimard, Ivers, Blais, Lavoie, & Boisvert, 2006), and the Social Interaction Phobia Scale (Duranceau, Peluso, Collimore, Asmundson, & Carleton, 2014). However, valid French versions of these questionnaires are not available for early adolescents and these instruments do not assess the lack of assertive behaviors, the fear of public speaking, and the difficulties with close and family members observed in socially anxious individuals. The detection of social anxiety and its behavioural difficulties in an early state is essential, especially for prevention. Therefore, the first purpose of this study was to translate and to examine the reliability and validity evidence drawn from the scores on the Questionnaire about Interpersonal Difficulties for Adolescents (QIDA; Inglés, 2009; Inglés, Hidalgo, & Méndez, 2005; Inglés, Torregrosa, Méndez, & Hidalgo, 2006) in a large sample of French adolescents from 11 to 18 years.

The QIDA is a 36-item self-report instrument designed to measure adolescents’ perceived interpersonal anxiety levels in a wide range of relationships with people of different ages, genders, levels of authority, and levels of intimacy and in several contexts including family, school, friends, opposite sex peers, and situations on the street, in shops and in public buildings. The QIDA provides important information for adolescents, educational and clinical psychologists, and researchers and can be used as a screening measure to identify social situations and relationships that adolescents find troublesome, thus allowing qualified professionals to ascertain the relevant target areas for social skills training programs (Inglés, 2009). Accordingly, the QIDA can be used as a counseling tool for advising adolescents and a remediation tool for addressing interpersonal difficulties. Furthermore, it can be used to develop prevention programs as well, in a variety of educational, clinical, and research settings. These contexts include schools, counseling and mental health centres, alcohol and other drug units for youth, correctional and social service agencies, and research centres (Inglés et al., 2006). In addition, since its initial development, the QIDA has been translated, using the back-translation method, into several languages, including Mandarin Chinese (Inglés, Marzo et al., 2008), Portuguese (Inglés, Castanheira, Ribeiro, & García-Fernández, 2008), Farsi (Shokri et al., 2010), Slovene (Zupancic, Inglés, Bajec, & Puklek, 2011) and Spanish as spoken in Colombia (Redondo, Delgado, Inglés, Hidalgo, García-Fernández, & Martínez-Monteagudo, 2013).

The psychometric properties of the QIDA are satisfactory among Spanish adolescents (Inglés et al., 2005; Inglés et al., 2008). Inglés et al. (2005) performed exploratory factor analysis (i.e., principal components factor analysis with oblimin rotation; PCFA) and CFA (maximum likelihood method) and found a correlated five-factor structure for scores on the QIDA for a large Spanish adolescent sample. Assertiveness (AS) consists of 16 items about difficulty or anxiety in social situations that differ in terms of potential danger such as making complaints, the defending of one’s rights and interests, rejecting unreasonable requests, and asking service staff (e.g., waiters and shop assistants) and strangers on the street for information. Heterosexual Relationships (HR) consists of 7 items that address difficulty or anxiety in heterosexual relationships (e.g., having a date or giving compliments). Public Speaking (PS) consists of 5 items that describe situations that put the adolescent in front of a large group of people or an audience. Family Relationships (FR) consists of 4 items about difficulty or anxiety when asserting oneself in a family environment. Finally, Close Friendships (CF) consists of 4 items about difficulty or anxiety expressing thanks and apologising to close friends, or handling criticism from them. The highest correlations (> .50) were obtained between the AS, HR, and PS factors, whereas the lowest correlations (< .30) were obtained between the FR factor, and the HR and PS factors. In later studies using CFA (maximum likelihood estimation), the correlated five-factor structure of scores on the QIDA was supported among Chinese (Inglés, Marzo et al., 2008), Iranian (Shokri et al., 2010), Slovene (Zupancic et al., 2011) and Colombian (Redondo et al., 2013) adolescents, whereas a correlated four-factor structure on the QIDA was found among Portuguese (Inglés, Castanheira et al., 2008) adolescents.

Inglés et al. (2005) reported satisfactory reliability of the QIDA, with a Cronbach’s alpha coefficient of .90 for the QIDA total scale and values ranging from .57 (Close Friends) to .85 (Heterosexual Relationships) for the five subscales. Similar Cronbach’s alphas were found for adolescents in different countries (e.g., China, Portugal, Iran, Slovenia, and Colombia), ranging from .57 to .92 for the five subscales and from .89 to .93 for the total scale.

There is also substantial support for the construct validity of the QIDA. Inglés et al. (2005) found positive and statistically significant correlations between interpersonal difficulties (QIDA total) and social anxiety as well as the other symptoms of anxiety that were self-reported such as fear of public speaking and neuroticism traits, whereas QIDA scores correlated negatively with security of public speaking and extraversion. Construct validity of the QIDA was also supported by marked differences in QIDA scores for adolescents with and without social phobia. Inglés et al. (2003) found positive and statistically significant correlations between the QIDA and the Social Anxiety, Assertiveness, and Making Friends subscales from the List of Social Situation Problems (LSSP) in a sample of Spanish high school students.

However, it has been observed that the relations between QIDA subscales and social anxiety tests vary as a function of the area assessed in each subscale. Thus, the magnitude of the correlations between the Assertiveness, Public Speaking, and Heterosexual Relationships subscales and symptoms of social anxiety is moderate to high, whereas the correlations are significant but low for the Close Friends and Family Relationships subscales (Inglés et al., 2005; Shokri et al., 2010; Zhou, Xu, Inglés, Hidalgo, & La Greca, 2008; Zupancic et al., 2011). This finding is explained by the greater difficulties shown by socially anxious adolescents when using assertive strategies, establishing relationships, or speaking in front of people, and less impairments on relationships with family members and close friends (Alden, Regambal & Plasencia, 2014). In addition, empirical evidence also suggests construct validity of scores on the QIDA across cultures (Inglés, Castanheira et al., 2008; Shokri et al., 2010; Zhou, Xu, Inglés, Hidalgo, & La Greca, 2008; Zupancic et al., 2011).

Given the importance of having a psychometrically sound measure to assess interpersonal anxiety to identify adolescents with likely impairments in social interactions, such as a lack of assertive behaviors, fear of public speaking, and difficulties with close friends and parents, the first purpose of this study is to examine the reliability and validity evidence drawn from the scores on the QIDA among a sample of French adolescents.

Based on previous findings (Inglés et al. 2003, 2005; Redondo et al., 2013; Zupancic et al., 2011), the following hypotheses were tested:

1. First, the correlated five-factor structure and scale score reliability found by Inglés et al. (2005) will be replicated with the data of the French QIDA.
2. Second, QIDA subscale scores will correlate statistically significantly and positively with scores on the Social Anxiety Scale for Adolescents (SAS-A; La Greca & Lopez, 1998) as both instruments measure anxiety perceived by adolescents in social situations. However, according to previous studies (Inglés et al., 2005; Zhou et al., 2008; Zupancic et al., 2011), it is expected that the links with social anxiety will be higher for the AS, HR, and PS subscales scores than for the FR and CF subscales scores of the QIDA.
3. Third, scores on the QIDA subscales will correlate statistically significantly and positively with scores on the School Anxiety Inventory (SAI; García-Fernández, Inglés, Martínez-Monteagudo, Marzo, & Estévez, 2011). Considering that no previous studies on relation between QIDA subscale scores and SAI scores have been published, we consider this hypothesis to be an open research question.

## Method

### Participants

The research study took place in the Haute-Garonne department, a region of southern France. Two-stage random sampling was conducted throughout the department. In the first stage, four public secondary schools were randomly selected to represent the department in Toulouse city and the neighbouring municipalities of Gratentour, Saint Orens de Gameville, and Colomiers. Once the schools were selected, nine classes were selected randomly from each school in the second stage of the sampling. Due to the random sampling method, the socioeconomic status and ethnic compositions of the overall sample are assumed to be representative of the community.

The initial sample consisted of 1,124 high school students from ages 11 to 18. Of this total, 74 (6.6%) students were excluded from the study because their parents did not provide informed written consent, 24 (2.1%) students were excluded because they were foreign nationals with major flaws in their knowledge of French language, and 69 (6.1%) students were excluded because their answers were incomplete. The final sample was comprised of 957 students (464 boys and 455 girls) with a mean age of 14.48 years (*SD* = 1.85; range =11–18 years). Of the participants, 33.1% were between 11 and 13 years of age (16.7% boys and 16.4% girls), 33.3% were between 14 and 15 years of age (16.3% boys and 17% girls), and 33.5% were between 16 and 18 years of age (15.5% boys and 18.1% girls). A chi-square test evaluated gender and age differences in the distribution of adolescents, finding no statistically significant differences for the eight Age x Gender groups (χ^2^ = 1.25, *df* = 2, *p* = .53). The effect size was small (< .30) (Cohen, 1988), thus supporting the homogeneity of the distribution of the sample for gender and age groups (Cramer’s V = .04).

### Measures

#### Questionnaire about Interpersonal Difficulties for Adolescents (QIDA; Inglés et al., 2005)

The QIDA is a 36-item self-report measure developed to measure interpersonal difficulties during adolescence. There are separate versions for male and female respondents that are identical except for the gender of nouns and pronouns. Each item is rated on a five-point Likert scale according to the difficulty of each situation and social relationship (0 = *no difficulty* to 4 = *maximum difficulty*). The QIDA consists of a total score and five subscale scores: Assertiveness (AS), Heterosexual Relationships (HR), Public Speaking (PS), Family Relationships (FR), and Close Friends (CF). As scores increase, so do indications of interpersonal anxiety.

The French translation of the QIDA was established using the back-translation method (Hambleton & Kanjee, 1995). First, the original Spanish version was translated into French by a French interpreter having a university degree in Spanish language and knowledge of the Spanish culture. Once completed, the French translation was back-translated into Spanish by a native French translator with a degree in Spanish and knowledge of both cultures. The original version was then compared with the back translation, and translators made corrections to the final French translation. No items were eliminated or significantly changed during the translation process. The items in French language are included in Table 2.

#### Social Anxiety Scale for Adolescents (SAS-A; La Greca & Lopez, 1998)

The SAS-A is a self-report questionnaire that measures social avoidance, fears, and worries in social situations among adolescents. It contains 18 items that are self-statements and four filler items. Items are rated on a five-point Likert scale (1 = *not at all* to 5 = *all of the time*). The SAS-A includes three subscales: Fear of Negative Evaluation (FNE) consists of 8 items that assess fears, concerns, or worries regarding peers’ negative evaluations (e.g., “I worry about what others say about me”); Social Avoidance and Distress in New Situations (SAD-N) consists of 6 items that assess social avoidance and distress in new social situations or with unfamiliar peers (e.g., “I get nervous when I talk to peers I do not know very well”); and Social Avoidance and Distress-General (SAD-G) consists of 4 items that assess general social inhibition, distress, and discomfort (e.g., “I am quiet when I am with a group of people”). Items from each subscale are summed such that higher scores reflect greater social anxiety. The three-factor structure of the SAS-A has been supported in factor analytic studies (La Greca & Lopez, 1998; Zhou et al., 2008). The SAS-A and subscales of the SAS-A demonstrated good Cronbach’s alpha and test–retest reliability and good construct validity.

In the current study, the SAS-A was translated into French language using the back-translation method (Hambleton & Kanjee, 1995). Alpha coefficients in this sample were adequate to good for SAD-General (.77), SAD-New (.72), FNE (.87), and the overall SAS-A score (.88).

#### School Anxiety Inventory (SAI; García-Fernández et al., 2011)

The SAI is designed to assess the situations and responses of school anxiety for adolescents. Items are answered on a Likert-type scale (0 = *never* to 4 = *always*). Exploratory and confirmatory factor analyses yielded four school situational factors and three response system factors (García-Fernández et al., 2011). The situational factors are Anxiety about Academic Failure and Punishment (AAFP; 8 items), which describes situations of school failure and punishment (e.g., “being sent to the head teacher”); Anxiety about Aggression (AA; 6 items), which describes situations of anxiety derived from suffering physical or verbal aggression by peers (e.g., “being insulted or threatened”); Anxiety about Social Evaluation (ASE; 5 items), which describes social fears related to public speaking (e.g., “speaking to the class”), and Anxiety about Academic Evaluation (AAE; 4 items), which describes situations where academic competence is assessed (e.g., “taking a written exam”). The response system factors are Cognitive Anxiety (CA; 9 items), which assesses thoughts and feelings about different school situations (e.g., “I am afraid of making mistakes”); Behavioural Anxiety (BA; 5 items), which assesses observable behaviours (e.g., “my voice trembles”); and Psychophysiological Anxiety (PA; 5 items), which assesses the reaction of the nervous system in several school situations (e.g., “I get nauseous”). García-Fernández et al. (2011) found evidence that supports the scale score reliability and validity using a sample of Spanish adolescents.

In this study, the SAI was translated into French language using the back-translation method (Hambleton & Kanjee, 1995). Cronbach’s alpha coefficients in the current study were good: .96 (SAI total), .95 (AAFP), .93 (ASE), .91 (AA), .85 (AAE), .88 (CA), .83 (BA) and .84 (PA).

### Procedure

The questionnaires were answered collectively and anonymously in the classroom, after obtaining informed consent from their parents. Research assistants informed the students that their participation was strictly voluntary and anonymous. The questionnaires were distributed with instructions and answer sheets, which were subsequently corrected by computer. Students were instructed to complete the identification data (sex, age, course, participant code, and school code). The instructions were read aloud and the importance of answering each question was emphasised. Research assistants supervised each administration, answered questions, and verified that respondents completed the test independently. The average administration times were 15 min (QIDA), 10 min (SAS-A), and 20 min (SAI).

### Data analyses

The data analytic plan proceeded in five steps. First, although sole reliance on confirmatory factor analysis (CFA) might be ill advised, experts on factor analysis generally recommend that exploratory factor analysis (EFA) precedes CFA when evaluating a new test or theory (e.g., Schmitt, 2011). Bearing in mind this recommendation, we engaged in a cross-validation of the EFA solution in a CFA approach to analyse the dimensional structure of the QIDA. To carry out the PAF and CFA analyses independently, the total sample (*N* = 957) was randomly divided in two subsamples: subsample 1: (*n*1 = 483; 235 boys and 248 girls) and subsample 2 (*n*2 = 474; 229 boys and 245 girls). The chi-square test and effect size supported the homogeneity of the distribution of the subsample 1 (χ^2^ = 1.51, *df* = 2, *p* = .47; Cramer’s V = .06) and subsample 2 (χ^2^ = .51, *df* = 2, *p* = .77; Cramer’s V = .03) for gender and age groups.

Second, a principal axis factoring (PAF) with promax rotation (Kaiser criterion) was conducted on the first subsample because of the assumption of correlated factors (Schmitt, 2011). Furthermore, research has shown that the Kaiser criterion for eigenvalues greater than 1 can either underestimate or overestimate the appropriate number of factors (Floyd & Widaman, 1995). The factorial solution, therefore was also determined using the scree plot method. Only items with a loading of .30 or higher were deemed important and used when interpreting the factors extracted (Child, 2006).

Third, CFA (robust maximum likelihood) was conducted on the second subsample to test the model obtained in the PAF and the model obtained for Inglés et al. (2005). For this analysis, we examined the normality or distribution of the QIDA items by obtaining univariate skewness, univariate kurtosis, and multivariate kurtosis values, following the procedures outlined by Finney and DiStefano (2006). To assess the adequacy of the models in confirmatory factor analyses, the robust comparative fit indexes Standardised Root Mean Square Residual (S-RMR), Comparative Fit Index (R-CFI), and the Root Mean Square Error of Approximation (R-RMSEA), were examined. A good fit is indicated by CFI values greater than .90 or close to .95 and standardised root mean square residual (SRMR) values less than .08. A root mean square error of approximation (RMSEA) value less than .06 indicates a good fit (Hu & Bentler, 1999).

Fourth, we computed internal consistencies of the QIDA scores on the entire sample using Cronbach’s alpha. Hunsley and Marsh (2008) established the following cut-offs for the Cronbach’s alpha for test scores: Adequate = values of .70 to .79; Good = values of .80 to .89; and Excellent = values greater than .90.

Fifth, to examine the construct validity of scores on the questionnaire, and bearing in mind the presence of severe non-normality in the distribution of scores on the QIDA, Spearman Rho correlation coefficients between QIDA score and the SAS-A and SAI scores were calculated, again on the entire sample.

## Results

### Validity evidence based on the internal structure of the QIDA scores

#### Principal Axis Factoring: Subsample 1

The factor solution was composed of 36 items grouped into five factors which were also obtained by Ingles et al. (2005), that accounted for 44.2% of the variance. Factor 1, Assertiveness (eigenvalue = 8.47), accounted for 23.52% of the variance and included 16 items about making complaints, defense of one’s rights and interests, rejecting unreasonable requests, and asking service staff, and strangers. Factor loading ranged from .70 to .31 (*M* = .46). Factor 2, Heterosexual Relationships (eigenvalue = 2.37), accounted for 6.59% of the variance and was composed of 7 items about heterosexual relationships. Factor loadings ranged from .84 to .56 (*M* = .75). Factor 3, Public Speaking (eigenvalue = 1.94), accounted for 5.40% of the variance and comprised 5 items referring to situations in which the adolescent has to act in front of a large group of people or an audience. Factor loading ranged from .76 to .60 (*M* = .69). Factor 4, Family Relationships (eigenvalue = 1.79), accounted for 4.98% of the variance and was composed of 4 items about assertiveness in the family environment. Factor loading ranged from .69 to .61 (*M* = .65). Factor 5, Close Friends (eigenvalue = 1.34), accounted for 3.71% of the variance and was composed of 4 items about assertiveness in interactions with friends. Factor loading ranged from .74 to .31 (*M* = .53).

The correlations between factors of the QIDA were statistically significant (*p* < .001) and moderate for AS-HR (–.41), HR-PS (–.37), AS-PS (.31), AS-CF (–.27), PS-CF (.21), and HR-CF (.20), subscales, low for FR-AS (.19), and FR-PS (.11) (Cohen, 1988), and non-significant for the FR-HR (.08) subscales.

#### Confirmatory factor analyses: Subsample 2

Univariate skewness and univariate kurtosis values indicated that the data were univariate non-normal. The range of values for univariate skewness was –.27 (Item 36) to 5.65 (Item 22). The range of values for univariate kurtosis was –1.37 (Item 21) to 38.10 (Item 22). We also found evidence of multivariate non-normality. The value of Mardia’s normalised multivariate kurtosis for all items of the QIDA was 76.04, which supports the presence of severe non-normality in the distribution of scores on the QIDA. Therefore, based on recommendations of Finney and DiStefano (2006), we used the Satorra-Bentler chi-square (SBχ^2^) scaling method and the robust ML estimation method for confirmatory factor analysis.

Three factor models were tested: the null model (Model 1), which assumes maximum independence among items (i.e., a model without a factor structure); the one-factor model (Model 2), which assumes that all items load on one factor (i.e., interpersonal anxiety); and the five-factor model (Model 3), which is based on the results of Inglés et al. (2005) and allows for intercorrelations among the five factors.

An overlap of content between Items 36 and 21 for the HR factor (items assess difficulties in situations to ask for a date), and possible content overlap between Items 12 and 22 of the CF factor (both items assess difficulties in thanking friends or helping them) were found. The five-factor model was subsequently re-specified and re-estimated with these two error covariances included (Model 4). This re-parameterisation resulted in a further slight improvement in model fit.

Following the Lagrange multiplier test in EQS 6.1 (Byrne, 2006), the SBχ^2^ modified test revealed that the best fit overall was the correlated five-factor model with the correlated errors (see Table 1). This model (Model 4) presented reasonable values in all indexes (S-RMR = .056; RCFI = .90; R-RMSEA = .037; 90% confidence interval = .033–.041). Furthermore, Model 4 represented a statistically significant improvement over Model 1 (χ^2^ (48) = 2278.41; *p* < .001) the Model 2 (χ^2^ (12) = 392.89; *p* < .001) and Model 3 (χ^2^ (4) = 7.11; *p* < .05).

**Table 1:** Fit statistics for confirmatory factor models.

Model	S-Bχ^2^	df	*p*	S-RMR	R-CFI	R-RMSEA

Null (Model 1)	4261.24	630	.000			.000
One latent variable (Model 2)	1871.66	594	.000	.077	.648	.067 (.064–.071)
Five latent variables (Model 3)	1074.92	584	.000	.069	.865	.042 (.038–.046)
Five latent variables (Model 4) with correlated errors: (HR: Items 36 and 21, CF: Items 12 and 22)	958.71	582	.000	.056	.900	.037 (.033–.041)

Table 2 shows that all factor loadings for latent variables assessed by confirmatory factor analysis were acceptable (> .30). All correlations between factors of the QIDA were statistically significant (*p* < .001) and high for AS–PS (.61), AS–CF (.59), AS–HR (.58), PS–CF (.57), HR–PS (.51), HR–CF (.50) and FR–CF (.50) subscales, and moderate for FR–AS (.32), FR–PS (.26), and FR–HR (.24) subscales (Cohen, 1988).

**Table 2:** Descriptive statistics, and factor loadings for the latent variables on the French version of the QIDA on the CFA (male version).

Items	Statement: As-tu des difficultés pour…	Factor loadings	Descriptive statistics	
			*M*	*SD*

Assertiveness				
1	dire à la caissière du supermarché qu’elle t’a rendu trois euros de moins?	.49	.50	.84
4	demander au serveur qu’il s’occupe de toi parce que tu es arrivé le premier?	.62	1.80	1.45
6	dire à un inconnu qui essaie de passer devant toi au cinéma de se mettre au bout de la file d’attente?	.64	1.25	1.28
10	te plaindre au serveur quand il te sert de la nourriture ou une boisson « en mauvais état »?	.61	1.50	1.30
13	demander à un inconnu qu’il éteigne sa cigarette parce que ça te gêne?	.50	1.86	1.41
14	vendre des billets de loterie dans la rue pour un voyage scolaire?	.34	1.14	1.30
15	demander à un serveur lorsque tu hésites sur le menu?	.52	.74	1.03
17	demander ton chemin à un inconnu quand tu te perds dans un quartier que tu ne connais pas?	.47	.79	1.12
18	demander au guichet de ta banque ou caisse d’épargne ce qu’il faut faire pour ouvrir un livret jeune?	.44	.87	1.08
19	dire à un parent (grands-parents, oncles...) que ses mauvaises plaisanteries te gênent?	.40	1.34	1.36
24	dire à un voisin que le bruit qu’il fait t’empêche de travailler?	.44	1.14	1.20
28	ramener un CD défectueux au magasin où tu l’as acheté?	.45	.82	1.14
29	dire « non » à un(e) ami(e) qui te demande de lui prêter ton vélo ou ton scooter?	.42	1.13	1.25
33	dire « non » à quelqu’un qui te demande de l’argent dans la rue?	.33	.50	.97
34	demander à un inconnu qu’il t’aide si tu tombes de ton vélo ou de ton scooter?	.58	1.36	1.34
35	demander à un serveur qu’il t’échange le coca qu’il t’a mal servi contre un jus d’orange?	.65	1.61	1.35
Heterosexual relationships				
2	faire des compliments à une fille qui t’intéresse?	.78	1.69	1.29
8	commencer une conversation avec une fille de ton âge que tu ne connais pas à l’arrêt d’autobus?	.64	1.85	1.37
11	dire à une fille qu’on vient de te présenter que ce qu’elle porte te plait?	.56	1.24	1.28
21	inviter une fille au cinéma?	.75	1.95	1.46
26	t’approcher et te présenter à une fille qui te plaît?	.82	2.14	1.36
32	commencer une conversation avec une fille qui te plaît?	.80	1.48	1.29
36	demander un rendez-vous à une fille?	.81	2.25	1.48
Public Speaking				
3	exposer en classe un travail que tu as préparé?	.59	1.34	1.15
5	exprimer ton opinion dans une réunion d’élèves quand tu n’es pas d’accord avec ce qu’on dit?	.67	.89	1.12
7	demander au professeur en classe de reprendre son explication lorsque tu ne comprends pas quelque chose?	.48	.75	1.03
16	être volontaire pour aller au tableau même si tu as préparé la leçon?	.49	1.48	1.36
20	exprimer ton point de vue devant tes camarades de classe?	.71	1.01	1.19
Family Relationships				
9	donner ton opinion à tes parents lorsque tu n’es pas d’accord avec eux?	.59	.24	.66
23	te défendre quand tes parents t’accusent de quelque chose que tu n’as pas fait?	.72	.20	.61
25	te défendre quand ton frère / ta sœur t’accuse de lui avoir abîmé quelque chose (livre, vêtement…)?	.30	.15	.47
31	te plaindre à tes parents quand ils t’interdisent d’aller à une sortie scolaire?	.33	.35	.78
Close Friendships				
12	remercier tes ami(e)s quand ils/elles te défendent?	.35	.15	.53
22	remercier un(e) ami(e) qui t’aide à faire tes devoirs?	.35	.11	.46
27	demander des excuses à un(e) ami(e) avec qui tu t’es disputé?	.47	1.03	1.17
30	défendre ton ami(e) quand on le/la critique?	.47	.27	.59

### Reliability evidence of scores on the QIDA

The Cronbach’s alpha estimates (and 95% CI) were .88 (AS; .87–.89), .90 (HR; .89–.91), .75 (PS; .73–.77), .74 (FR; .71–.76), and .72 (CF; .70–.74). With Cronbach’alpha values higher than .70 for all subscales, the QIDA scores demonstrated evidence of excellent (HR), good (AS) and adequate (PS, FR, and CF) reliability on their subscales.

### Validity evidence based on relations of the QIDA and the social anxiety and school anxiety self-reports

To examine the construct validity of scores on the QIDA, and bearing in mind the presence of severe non-normality in the distribution of scores on the QIDA, Spearman Rho correlation coefficients were calculated between the QIDA total and subscale scores, between the SAS-A total and the subscale scores, and between that SAI total and the subscale scores (see Table 3). The QIDA subscale scores correlated positively and statistically significantly with all measures of social anxiety (SAS-A) and school anxiety (SAI) with the exception of the correlation between CF (QIDA subscale) and AAFP (SAI subscale), which did not exceed the established criteria of statistical significance. The associations between the QIDA scores and SAI and SAS-A scores varied according to the scales of the QIDA. Stronger relations with social anxiety and school anxiety were also shown for the AS, HR, and PS subscales of the QIDA, whereas the associations with difficulties in the family and close friend domains were low. Thus, moderate and high associations (*r_s_* = .31–.54) of fear of negative evaluation and social avoidance and distress (SAD-N and SAD-G) with difficulties in public speaking, assertiveness, and heterosexual relationships were revealed, whereas the correlations between the social anxiety subscales and FR and CF were low. This provides evidence for the construct validity of scores on the QIDA.

**Table 3:** Spearman Rho Correlations of QIDA scales with social anxiety and school anxiety. *Note.* ** *p* < .001, * *p* < .05. AS = Assertiveness, HR = Heterosexual Relationships, PS = Public Speaking, FR = Family Relationships, CF = Close Friendships, AAFP = Anxiety about Academic Failure and Punishment, ASE = Anxiety about Social evaluation, AA = Anxiety about Aggression, AAE = Anxiety about Academic Evaluation, CA = Cognitive Anxiety, BA = Behavioural Anxiety, PA = Physiological Anxiety, Total SAI = School Anxiety Inventory, FNE = Fear of Negative Evaluation, SAD-N = Social Avoidance and Distress in New Situations, SAD-G = Social Avoidance and Distress in General, Total SAS-A = Social Anxiety Scale for Adolescents.

	AS	HR	PS	FR	CF

AAFP	.30**	.31**	.25**	.17*	.08
ASE	.39**	.44**	.69**	.17*	.23**
AA	.47**	.49**	.31**	.23**	.27**
AAE	.33**	.44**	.49**	.20**	.25**
CA	.43**	.45**	.44**	.22**	.20**
BA	.39**	.44**	.51**	.24**	.24**
PA	.34**	.41**	.49**	.18*	.24**
Total SAI	.43**	.46**	.49**	.21**	.26**
FNE	.32**	.33**	.31**	.18*	.26**
SAD-N	.39**	.54**	.45**	.21**	.25**
SAD-G	.35**	.41**	.43**	.24**	.26**
Total SAS-A	.42**	.50**	.45**	.23**	.30**

Similar results were obtained for the correlations between school anxiety and the QIDA subscales. The SAI total and the subscale scores correlated moderately with the AS and HR subscale scores of the QIDA and, to a lesser degree, with the FR and CF subscale scores of the QIDA. Finally, the associations between difficulties in public speaking and school anxiety ranged from low to high (*r_s_* = .25–.69) depending on the school situations wherein anxiety appeared.

## Discussion

The purpose of this study was to examine the reliability and validity evidence drawn from the scores on the QIDA in a sample of French adolescents. The main results of the study provide support for the reliability and validity of scores on the QIDA and confirm the good psychometric properties of this self-report measure to assess interpersonal anxiety in adolescents (Tulbure et al., 2012).

Although Inglés et al. (2005) applied a PCFA with oblimin rotation, this study applied a PAF with promax rotation because many researchers have shown that PAF presents the most psychometric advantages (see Costello & Osborne, 2005; Kaplan, 2009; Schmitt, 2011; Widaman, 2007, for a review). In this case, the PAF replicated the factor solution found by Inglés et al. (2005) in a sample of Spanish adolescents. This solution comprised five factors that accounted for 44.2% of the variance.. Furthermore, confirmatory factor analysis replicated the same correlated five-factor structure, thus supporting the first hypothesis. Although the intercorrelations among the subscale scores of the QIDA (mean *r* = .47) suggest that the five domains are moderately related, they represent different aspects of interpersonal anxiety, particularly in associations between family relationships and relations with the opposite sex and impairment related to public speaking and assertiveness. Similar results were obtained in studies conducted in different countries (e.g., Spain, Slovenia, Colombia, and Portugal), thus supporting that conflicts with parents are relatively less related with fear of public speaking, difficulties relating to assertiveness and heterosexual interactions.

Adequate reliability was found for the French version of QIDA. According to the rating criteria proposed by Hunsley and Marsh (2008), the internal consistency coefficients (Cronbach’s alpha) were adequate to good for the AS, HR, PS, FR, and CF subscales of QIDA. These results were comparable to findings for the original QIDA (Inglés et al., 2005), and the Chinese (Inglés, Marzo et al., 2008), Slovene (Zupancic et al., 2011), and Colombian versions (Redondo et al., 2013), thereby providing support for the first hypothesis. Despite the positive findings of this study that supported the validity of the AS, HR and PS subscales, it would be suitable to assess the validity of the CF and FR subscales through more specific questionnaires for anxiety in social relationship with friendship and family, for example, the Making Friends subscale as found in the List of Social Situations Problems (LSSP; Spence & Liddle, 1990).

Consistent with the second hypothesis, interpersonal difficulties were highly to moderately correlated with the SAS-A total score and the SAS-A subscales. This result supports the clear link between impairments in social interactions and the development of social anxiety, social fears, and distress in social situations obtained in several previous studies (Inglés et al., 2003; Inglés et al., 2005; Inglés, Marzo et al., 2008; Zhou et al., 2008; Zupancic et al., 2011). In addition, moderate and high associations of fear of negative evaluation and social avoidance and distress (SAD-N and SAD-G) with difficulties in public speaking, assertiveness, and heterosexual relationships were revealed, whereas the correlations between the social anxiety subscales and FR and CF of the QIDA were low. This finding confirms that individuals with social anxiety tend to show greater difficulties in assertiveness, establishing relationships, or speaking in front of people, but their impairments on relationships with family members and close friends are less noticeable (Alden et al., 2014; Inglés et al., 2005; Zhou et al., 2008; Zupancic et al., 2011). Furthermore, greater interpersonal difficulties were associated with higher school anxiety in the three response systems (i.e., cognitive, behavioural and physiological anxiety) and in four school anxiety situations (i.e., academic failure and punishment, social evaluation, aggression, and academic evaluation), providing support for the third hypothesis. The results are also consistent with previous findings (e.g., Kearney & Albano, 2004; Miller et al., 2011; Morris & March, 2004) that suggest high comorbidity between interpersonal anxiety and academic anxiety and relevance of social skills training in anxiety treatment programs to mitigate the interpersonal difficulties in anxious students.

This study has some limitations that should be resolved in future research. First, although the results demonstrate adequate evidence of Cronbach’s alpha reliability and construct validity, it would be important to investigate test-retest reliability (i.e., temporal stability), predictive validity (i.e., diagnostic utility for detecting socially anxious adolescents) and discriminant validity (i.e., correlations with different assessment instruments). Additionally, to accumulate further evidence on construct validity, it would be useful to analyse the measurement invariance of the QIDA across gender and ethnic groups in French adolescents. Furthermore, the QIDA should be analysed with respect to its ability to detect improvements in social functioning resulting from treatment programs in samples of French adolescents. The QIDA has separate versions for male and female respondents that are identical except for the gender of nouns and pronouns. However, some of the items on this questionnaire may not be relevant to certain population groups (e.g., sexual minority youth and adolescents that do not have parents). Accordingly, future research should examine reliability and validity evidence of the QIDA scores in groups with different sexual orientations (e.g., homosexually oriented adolescents) and family types (e.g., adolescents with a single mother or father). Despite these limitations, the results of the present study suggest that the QIDA is a psychometrically sound measure for assessing interpersonal anxiety in adolescents and support the use of scores on the QIDA in the French-speaking adolescent population.

## Competing Interests

The authors declare that they have no competing interests.
